# Unusual dermoscopic patterns of basal cell carcinoma mimicking melanoma

**DOI:** 10.1111/exd.14533

**Published:** 2022-02-06

**Authors:** Eleonora Di Matteo, Riccardo Pampena, Maria A. Pizzichetta, Elisa Cinotti, Johanna Chester, Shaniko Kaleci, Marco Manfredini, Stefania Guida, Emi Dika, Elvira Moscarella, Aimilios Lallas, Zoe Apalla, Giuseppe Argenziano, Jian L Perrot, Linda Tognetti, Michela Lai, Carmen Cantisani, Vincenzo Roberti, Diletta Fiorani, Carlotta Baraldi, Leonardo Veneziano, Chryssoula Papageorgiou, Silvana Ciardo, Pietro Rubegni, Iris Zalaudek, Annalisa Patrizi, Caterina Longo, Luca Bianchi, Giovanni Pellacani, Francesca Farnetani

**Affiliations:** ^1^ Dermatologic Unit Department of Systems Medicine University of Rome Tor Vergata Rome Italy; ^2^ Department of Dermatology University of Modena and Reggio Emilia Modena Italy; ^3^ Centro Oncologico ad Alta Tecnologia Diagnostica Azienda Unità Sanitaria Locale‐IRCCS di Reggio Emilia Reggio Emilia Italy; ^4^ Division Medical Oncology and Preventive Oncology National Cancer Institute Aviano Italy; ^5^ Dermatology Clinic Maggiore Hospital University of Trieste Trieste Italy; ^6^ Department of Medical Surgical and Neurological Science Dermatology Section S. Maria Alle Scotte Hospital University of Siena Siena Italy; ^7^ Dermatology IRCCS di Policlinico Sant'Orsola Hospital Bologna Italy; ^8^ Dermatology Section Department of Experimental Diagnostic and Specialty Medicine DIMES University of Bologna Bologna Italy; ^9^ Dermatology Unit University of Campania Luigi Vanvitelli Naples Italy; ^10^ First Department of Dermatology School of Medicine Aristotle University Thessaloniki Greece; ^11^ Second Department of Dermatology School of Medicine Aristotle University Thessaloniki Greece; ^12^ Department of Dermatology University Hospital of Saint‐Etienne Saint‐Etienne France; ^13^ Dermatology Clinic Department of Clinical Internal Anesthesiological and Cardiovascular Sciences Sapienza University of Rome Rome Italy

**Keywords:** basal cell carcinoma, dermoscopy, malignant melanoma, non‐invasive diagnosis, non‐invasive techniques

## Abstract

**Background:**

Basal cell carcinoma can simulate melanoma and specific dermoscopic criteria have not yet been defined in a large cohort.

**Objective:**

To identify dermoscopic “trump” characteristics for differential diagnosis, identify cluster groups and assess the clinical impact of this study's findings.

**Methods:**

Retrospective, multicentric comparative study of atypical, non‐facial basal cell carcinoma (≥1 seven‐point checklist criteria) and melanoma (with at least one BCC criteria) at dermoscopy. Observed dermoscopic features were used to develop a proposed score. Lesion clusters were defined with hierarchical analysis. Clinical impact was assessed with a blinded reader study following this study's results.

**Results:**

A total of 146 basal cell carcinoma and 76 melanoma were included. Atypical vascular pattern was common to most lesions (74.5%). Twelve trump features were included in the proposed score (sensitivity 94.1% and specificity 79.5%). Cluster analysis identified 3 basal cell carcinoma and 3 melanoma clusters. Findings improved overall diagnostic accuracy and confidence (26.8% and 13.8%, respectively; *p* < 0.001).

**Conclusions:**

These findings support the notion that atypical vascular pattern should be considered a shared feature of both melanoma and atypical basal cell carcinoma. Our proposed score improves diagnostic accuracy and confidence. Absence of pigmented features was associated with lower diagnostic accuracy and confidence.

**Abbreviations**
BCC: basal cell carcinoma
MM: malignant melanoma
RCM: reflectance confocal microscopy

## 1 INTRODUCTION

Basal cell carcinoma (BCC) is the most common malignant skin cancer and is characterized by higher incidence in fair‐skin individuals, slow growth and rare cases of metastasis.^1^ The most important risk factor for the development of BCC is exposure to ultraviolet light, as confirmed by its frequent onset in photoexposed areas.^2^

In addition to clinical examination, dermoscopy has significantly improved BCC diagnostic accuracy, and criteria have been widely described revealing high sensitivity and specificity.^3^, ^4^ However, some BCCs may exhibit equivocal dermoscopic patterns, without typical dermoscopic criteria, simulating other malignant skin tumors, such as malignant melanoma (MM).^5^, ^6^, ^7^ Non‐facial BCCs simulating MMs have been described in literature with an array of predominant parameters, including atypical network, blue‐white veil, irregular dots/globules, irregular streaks and atypical vascular pattern.^8^, ^9^, ^10^ Presence of irregular streaks, linear irregular and atypical vessels specifically for lower limb BCCs has been reported.^11^, ^12^

A previous study of unselected, consecutive facial and non‐facial excised BCCs with histopathological diagnosis was retrospectively evaluated to investigate the variability and significance of dermoscopic features. Dermoscopic features suggestive of melanocytic lesions were observed in 40.6% of BCCs and significantly increased in heavily pigmented BCCs.^10^ A large, descriptive study of selected, atypical non‐facial BCCs with dermoscopic melanocytic features however is lacking. Differential diagnosis with dermoscopy would improve patient management with correct timing of excision and local treatment.

The current study aims to identify “trump” dermoscopic features in a selected cohort of atypical, non‐facial BCCs with melanocytic features (at least 1 feature of the seven‐point checklist^13^) compared to atypical, non‐facial MMs with at least 1 feature of BCC, for the development of a score useful for differential diagnosis. Secondary objectives include the identification of BCC and MM subtypes and the assessment of this study's clinical impact with a reader study of diagnostic accuracy and confidence.

## 2 MATERIALS AND METHODS

We performed a retrospective, multicentric observational study for excised atypical BCC lesions showing at least one of the revised seven‐point checklist criteria at dermoscopy, compared with excised atypical MM lesions showing at least one BCC criteria at dermoscopy. This study was approved by the Ethics committee of Modena (protocol No 169/17).^14^

Cases were selected from dedicated databases at collaborating centres: 7 Italian (Modena, Reggio Emilia, Bologna, Siena, Naples, Rome and Aviano), 1 Greek (Thessaloniki) and 1 French (Saint‐Etienne). Non‐facial lesions, registered between January 2010 and December 2018, were included. Patient age at diagnosis and gender, lesion body site, dermoscopic images and presurgical diagnoses were collected. All images were acquired with a polarized light dermatoscope.

One dermatologist (E.D.M) assessed clinical images for skin phototype (Fitzpatrick type I/II/III/IV), actinic damage in surrounding skin, ulceration, scales, crust, shiny surface, hair, skin markings, asymmetry, border irregularity, colour variegation (>2 colours) and diameter >6 mm. Two dermatologists (M.A.P/C.C, E.D.M) with substantial dermoscopy experience, independently evaluated dermoscopic images for the presence of BCC and MM specific and common criteria, outlined in Table S1.

Cluster analysis was used to identify BCC and MM subgroups, to describe the most obvious features observed among this heterogeneous lesion cohort. According to the most obvious characteristics for each subgroup, descriptive names were arbitrarily given.

The identification of “trump” dermoscopic features was identified based on multivariable analysis (outlined below). A score of 12 dermoscopic features was developed.

Evaluator diagnostic accuracy and confidence were performed by two independent evaluators (M.M, S.G), blinded to histology diagnoses. They were asked to diagnose each lesion's dermoscopy image (BCC or MM) and indicate a level of diagnostic confidence on a VAS scale (1 = not confident at all, 5 = very confident), considered T0.

The two independent evaluators were shown the score and asked to diagnose each image again (T1) with a level of diagnostic confidence, considering the 12 trump features.

### 2.1 Statistical analysis

Descriptive statistics and complete case analysis were used for all comparisons between groups. Pearson's χ2 test and Fisher's exact test were used to compare categorical variables in univariate analysis. A hierarchical cluster analysis was performed to identify potential homogeneous subgroups of BCC and MM lesions. All variables were included, and the optimal number (k) of clusters was determined with the Calinski and Harabasz stopping method^15^: The largest pseudo‐F value indicates the most distinct clustering. After selecting the optimal number of clusters, the cluster characteristics were analysed with the χ2 test. Dendrograms graphically present the grouping of observations at various levels of (dis)similarity. The association between parameters and outcome was assessed using logistic regression (with backward stepwise process). Multivariate logistic regression (stepwise selection method) was used to identify prognostic factors between groups. *p* < 0.05 defined variable inclusion into the model and “goodness of fit” was evaluated with Hosmer and Lemeshow test; data were expressed as odds ratio (OR) and 95% confidence interval (CI). The area under the curve (AUC), calculated by receiver operating characteristic (ROC) analysis, assessed predictability of MM diagnosis. All statistical analyses were performed using STATA^®^ software version 14 (StataCorp 2015; Stata Statistical Software: Release 14; StataCorp LP, College Station, TX, USA), and *p* < 0.05 was considered statistically significant.

## 3 RESULTS

### 3.1 Study population

A total of 146 BCCs and 76 MMs (222 patients, 444 dermoscopy observations) were included. BCC and MM were comparable for patient sex and lesion location, but the average patient age associated with MM diagnosis was significantly younger, as expected (*p* = 0.005). Clinical features were significantly different among the groups for all features, with the exception of actinic damage in surrounding skin and crust, see Table S2.

**FIGURE 1:**
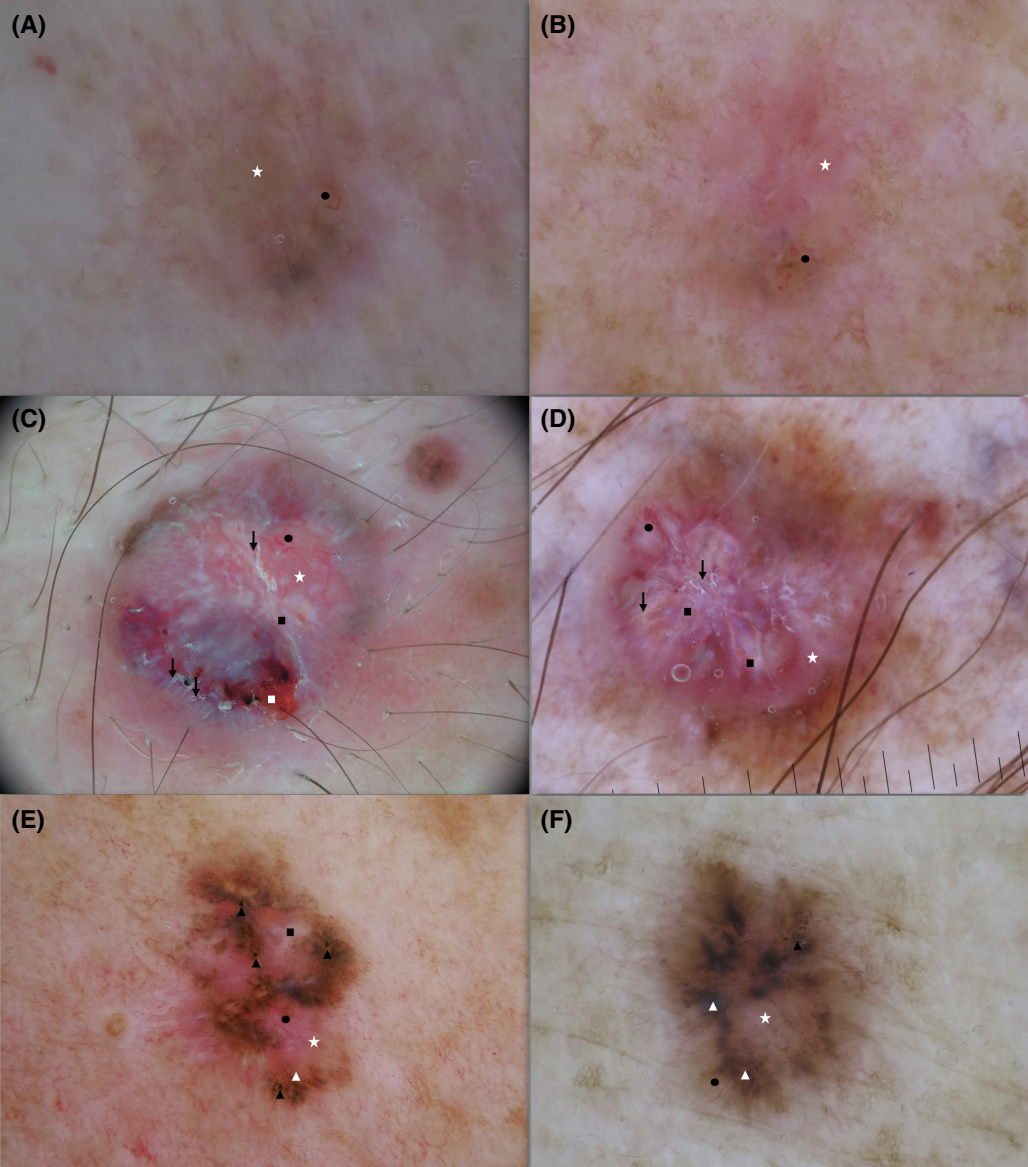
MM (a‐c‐e) and BCC (b‐d‐f) dermoscopic images: (a‐b‐c‐d‐e‐f) white‐red structureless areas (white star), (a‐b‐c‐d‐e‐f) atypical vascular pattern (black circle), (e‐f) multiple blue‐grey globules (black triangle), (c) ulceration (white rectangle), (c‐d‐e) regression structures (black rectangle), (c‐d) white streaks (arrows), (e‐f) maple leaf‐like areas (white triangle). MM, malignant melanoma; BCC, basal cell carcinoma

The most common dermoscopic features observed among the selected BCC lesions included MM‐associated atypical vascular pattern (70.9%), BCC‐associated superficial (short) fine telangiectasia (66.1%), multiple blue‐grey globules (61.6%) and MM‐ and BCC‐associated white‐red structureless areas (66.4%), see Table 1, Figure 1.

**TABLE 1:** Correlation of included dermoscopic features for all lesions (146 BCC and 76 MM lesions with double observations) and according to BCC and MM clusters

	All lesions			BCC Cluster Analysis			MM Cluster Analysis		
	Total, *n* (%)	BCC, *n* (%)	MM, *n* (%)	Hypo/Amelanotic MM‐like, *n* (%)	Pigmented‐BCC‐type, *n* (%)	Mixed, *n* (%)	Hypo/Amelanotic MM, *n* (%)	Pigmented‐MM‐type, *n* (%)	Pigmented‐BCC‐like. *n* (%)
Dermoscopic features	444 (100)	292 (65.8)	152 (34.2)	87 (29.8)	65 (22.3)	140 (47.9)	21 (13.8)	74 (48.7)	57 (37.5)
**MM‐associated (seven‐point checklist):**									
Atypical network	89 (20.0)	32 (11)	57 (37.5)***	3 (3.4)	11 (16.9)	18 (12.9)*	2 (9.5)	43 (58.1)	12 (21.1)***
Blue‐white veil	165 (37.2)	122 (41.8)	43 (28.3)**	40 (46.0)	35 (53.8)	47 (33.6)*	5 (23.8)	3 (4.1)	35 (61.4)**
Atypical vascular pattern	331 (74.5)	207 (70.9)	124 (81.6)*	83 (95.4)	15 (23.1)	109 (77.9)***	21 (100.0)	62 (83.8)	41 (71.9)*
Irregular dots/globules	209 (47.1)	110 (37.7)	99 (65.1)***	1 (1.1)	36 (55.4)	73 (52.1)***	4 (19.1)	45 (60.8)	50 (87.7)***
Irregular streaks	76 (17.1)	38 (13)	38 (25)**	0 (0.0)	24 (36.9)	14 (10.0)***	1 (4.8)	21 (28.4)	16 (28.1)**
Irregular blotches	127 (28.6)	59 (20.2)	68 (44.7)***	12 (13.8)	26 (40.0)	21 (15.0)***	2 (9.5)	30 (40.5)	36 (63.2)***
Regression structures	232 (52.3)	104 (35.6)	128 (84.2)***	10 (11.5)	33 (50.8)	61 (43.6)***	19 (90.5)	71 (96.0)	38 (66.7)***
**BBC‐associated:**									
Arborizing vessels	173 (39)	157 (53.8)	16 (10.5)***	60 (69.0)	4 (6.2)	93 (66.4)***	8 (38.1)	2 (2.27)	6 (10.5)***
Superficial (short) fine telangiectasias	273 (61.5)	193 (66.1)	80 (52.6)**	70 (80.5)	9 (13.8)	114 (81.4)***	17 (81.0)	47 (63.5)	16 (28.1)**
Blue‐grey ovoid nests	158 (35.6)	113 (38.7)	45 (29.6)	21 (24.1)	33 (50.8)	59 (42.1)**	10 (47.6)	2 (2.27)	33 (57.9)
Multiple blue‐grey globules	231 (52)	180 (61.6)	51 (33.6)***	27 (31.0)	46 (70.8)	107 (76.4)***	0 (0)	18 (24.3)	33 (57.9)***
In‐focus dots	142 (32)	115 (39.4)	27 (17.8)***	15 (17.2)	31 (47.7)	69 (49.3)***	1 (4.8)	8 (10.8)	18 (31.6)***
Maple leaf‐like areas	123 (27.7)	117 (40.1)	6 (3.9)***	8 (9.2)	47 (72.3)	62 (44.3)***	0 (0)	1 (1.4)	5 (8.8)***
Spoke‐wheel areas	59 (13.3)	55 (18.8)	4 (2.6)***	0 (0.0)	14 (21.5)	31 (22.1)***	0 (0)	1 (1.4)	3 (5.3)***
Concentric structures	80 (18)	77 (26.4)	3 (2)***	19 (21.8)	17 (26.2)	42 (30.0)	0 (0)	3 (4.1)	0 (0)***
Multiple small erosions	95 (21.4)	60 (20.5)	35 (23)	9 (10.3)	6 (9.2)	45 (32.1)***	17(81.0)	2 (2.7)	16 (28.1)
**MM‐ and BCC‐associated:**									
Ulceration	132 (29.7)	106 (36.3)	26 (17.1)***	64 (73.6)	14 (21.5)	28 (20.0)***	11 (52.4)	0 (0)	15 (26.3)***
White‐red structureless areas	335 (75.5)	194 (66.4)	141 (92.8)***	83 (95.4)	19 (29.2)	92 (65.7)***	21 (100.0)	73 (98.7)	47 (82.5)***
White streaks	260 (58.6)	147 (50.3)	113 (74.3)***	69 (79.3)	16 (24.6)	62 (44.3)***	17 (81.0)	63 (85.1)	33 (57.9)***

Abbraviations: MM, Malignant melanoma; BCC, Basal cell carcinoma.

* *p* < 0.05; ** *p* < 0.01; *** *p* < 0.001.

The most common dermoscopic features observed among the selected MM lesions were the same as those for BCC lesions (atypical vascular pattern [81.6%], superficial (short) fine telangiectasia [52.6%] and white‐red structureless areas [92.8%]). Additionally, MM‐associated regression structures (84.2%), and MM‐ and BCC‐associated white streaks (74.3%) were also very frequently observed, see Table 1, Figure 1.

The identification of “trump” dermoscopic features and their influence on final histopathological diagnosis was assessed with a multivariable analysis, outlined in Table S3. In order to assess the clinical applicability of our study, we shared these findings with the blinded evaluators. Overall, diagnostic accuracy and confidence levels improved between T0 and T1 by 27% (68.5% to 86.9%) and 14% (2.9 to 3.3), respectively, see Table S4.

A score, based on regression analysis and including 12 dermoscopic features, was then devised for the differential diagnosis of MM/BCC, see Table 2. We elected to use arbitrary multipliers, rather than exact odds ratios to maximize clinical applicability. The models’ sensitivity and specificity were 94.08% and 79.45%, respectively.

**TABLE 2:** Score for the diagnosis of MM/BCC based on dermoscopic features predictive of differential diagnosis

Dermoscopic Features	SCORE
Regression Structures	+3
Irregular Dots/Globules	+3
Irregular Blotches	+2
Irregular Streaks	+2
White‐red structureless areas	+1
White streaks	+1
Spoke‐wheel areas	−1
In‐focus dots	−1
Multiple blue‐grey globules	−1
Arborizing vessels	−2
Concentric structures	−3
Maple leaf‐like areas	−3
**Total (Cut‐off for MM >2/BCC ≤2)**	

Note

Sensitivity = 94.08%; Specificity = 79.45%.

### 3.2 Cluster analysis

A hierarchical cluster analysis of BCC and MM lesions identified 3 clusters for each lesion diagnostic group, see Table 1. Cluster groups were homogeneous for demographic characteristics and lesion location (data not shown).

**FIGURE 2:**
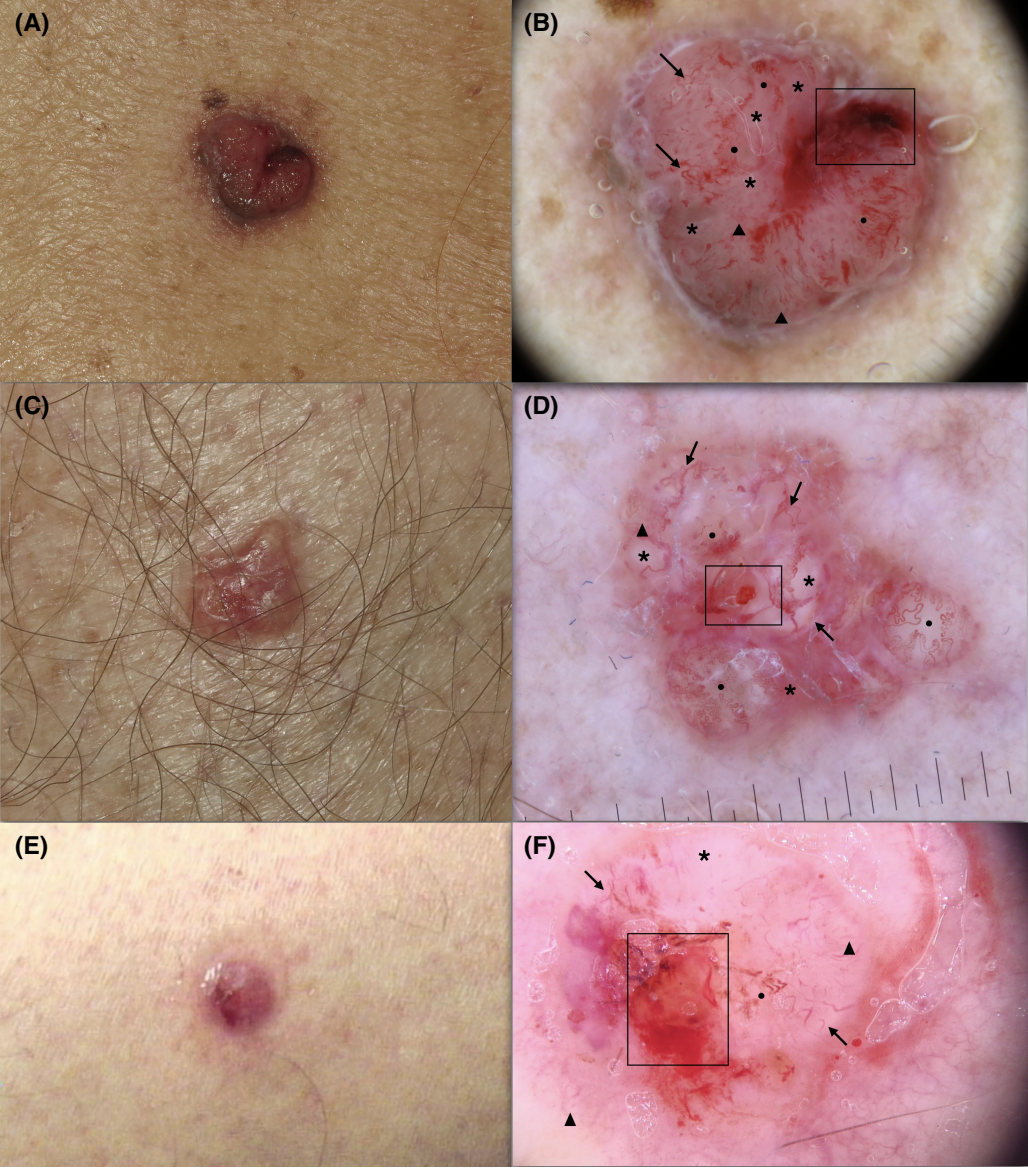
BCC lesions representative of the hypo/amelanotic MM‐like cluster: (a‐c‐e) clinical images;(b‐d‐f) white‐red structureless areas (asterisk), (b‐d‐f) atypical vascular pattern (black circle), (b‐d‐f) superficial (short) fine telangiectasias (triangle), (b‐d‐f) ulceration (rectangle), (b‐d‐f) some arborizing vessels (arrows). BCC, basal cell carcinoma

The BCC “hypo/amelanotic‐MM like” cluster included 44 lesions (30.1%), characterized principally by trunk and lower limbs lesion location and hypopigmented BCCs (data not shown). Both BCC and MM dermoscopic non‐pigment‐related criteria were observed in this cluster, with a predominance of MM criteria. In particular, white‐red structureless areas and atypical vascular pattern (95.4%, respectively) were significantly more frequently observed in this cluster (*p* < 0.001). Superficial (short) fine telangiectasia (80.5%), white streaks (79.3%) and ulceration (73.6%) were also frequently observed. Pigment‐related criteria were reported in a minority of cases, see Table 1, Figure 2.

**FIGURE 3:**
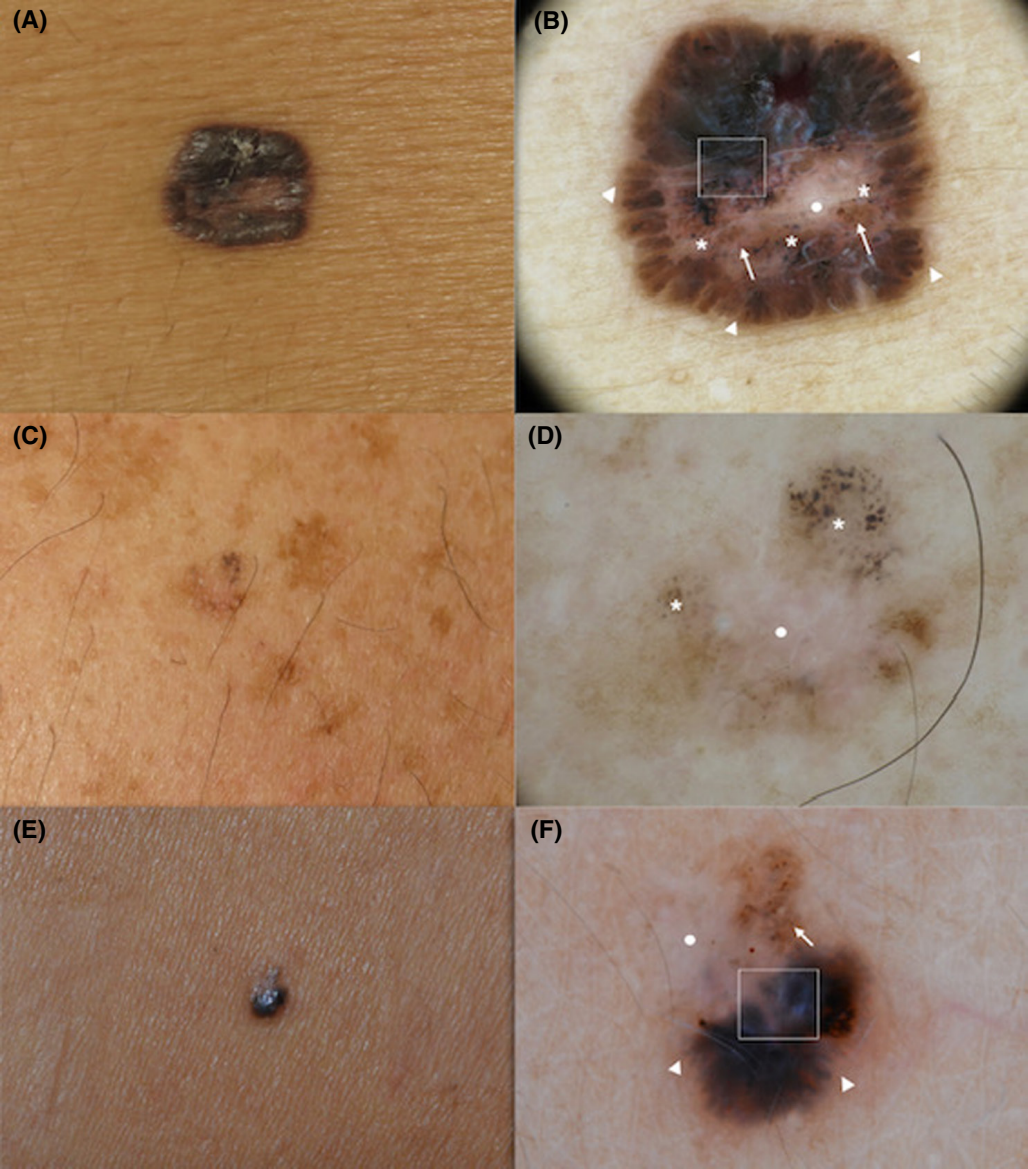
BCC lesions representative of the pigmented‐BBC‐type cluster: (a‐c‐e) clinical images; (b‐f) maple leaf‐like areas (triangle), (b‐d‐f) multiple blue‐grey globules (asterisk), (b‐d‐f) regression structures (white circle), (b‐f) blue‐white veil (rectangle) and (b‐f) some irregular dots/globules (arrows). BCC, basal cell carcinoma

The BCC “pigmented‐BCC‐type” cluster, included 33 lesions (22.6%), characterized by pigmented BCCs. Cases mainly displayed dermoscopic BCC specific criteria, such as maple leaf‐like areas (72.3%) and multiple blue‐grey globules (70.8%). Some MM specific pigment‐related criteria were also frequently observed, including irregular dots/globules, regression structures and blue‐white veil in over half of the lesions. Non‐pigment‐related features associated with MM diagnosis were less represented in this cluster compared to other clusters, see Table 1, Figure 3.

**FIGURE 4:**
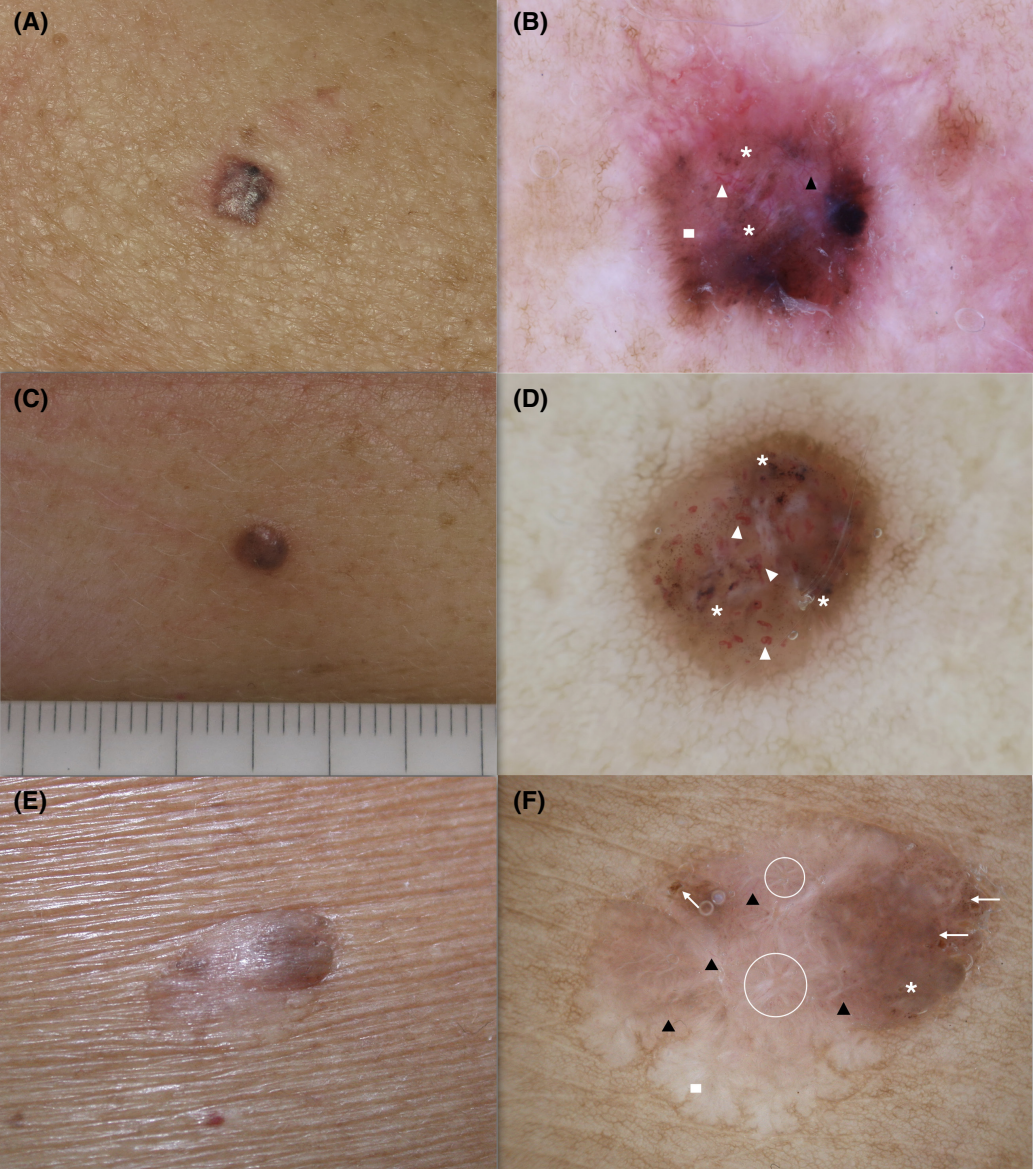
BCC lesions representative of the mixed cluster: (a‐c‐e) clinical images; (b‐f) superficial (short) fine telangiectasias (black triangle), (b‐d‐f) multiple blue‐grey globules (asterisk), (b‐d) atypical vascular pattern (white triangle), (b‐f) white‐red structureless areas (rectangle), (f) irregular dots/globules (arrows), (f) white streaks (circle). BCC, basal cell carcinoma

The largest BCC cluster “mixed” included 69 lesions (47.3%) and was mainly characterized by features correlated with both BCC and MM dermoscopic criteria. There was a predominance of vascular and pigmented patterns, including superficial (short) fine telangiectasia (81.4%), atypical vascular pattern (77.9%), multiple blue‐grey globules (76.4%), arborizing vessels (66.4%) and white‐red structureless areas (65.7%), see Table 1, Figure 4.

Lesions included in the first MM cluster “hypo/amelanotic MM” are comparable to the first BCC cluster “hypo/amelanotic‐MM‐like” given the limited presence of pigmented features, see Figure 1. The remaining MMs were characterized by pigmented features and a strong presence of MM‐associated features, with the main difference between these subgroups being the higher presence of BCC‐associated features in the third “pigmented‐BCC‐like” cluster. In the pigmented‐MM‐like subgroup, the almost exclusive BCC‐associated features observed were superficial (short) fine telangiectasia.

### 3.3 Clinical impact of study findings

The trump features were shared with the blinded, independent evaluators who were asked to evaluate the images again considering the 12 features identified. The second evaluations were then compared to their initial evaluations. Both overall diagnostic accuracy and confidence levels significantly improved (26.8% and 13.8%, respectively; *p* < 0.001), with the greatest improvement noticed in overall BCC diagnostic accuracy, see Table S4. Diagnostic accuracy and confidence levels for each cluster group were significantly improved (*p* > 0.05), with the exception of hypo/amelanotic MM lesions, which remained unchanged at T1. The most difficult BCC and MM clusters to diagnose with the lowest confidence levels were and remained hypo/amelanotic lesions.

## 4 DISCUSSION

In these selected, non‐facial BCC lesions mimicking MM at dermoscopy and MM lesions with BCC‐associated dermoscopy features, all features of the seven‐point checklist and the BCC‐associated features were observed, but at different frequencies.

Despite the age differences between the BCC and MM groups (as was expected^1^), the groups were homogeneous in sex and lesion location. However, according to clinical features, our evaluation confirms that BCC arises more frequently on phototype I/II and more often have a shiny surface compared to MM, while skin markings, hair and irregular borders and asymmetry are characteristics more frequently associated with MM diagnosis.

The most evident features of the seven‐point checklist observed among BCC clusters were atypical vascular pattern. This feature has also been reported by other authors among BCC lesions with atypical presentation.^10^, ^12^ However, patient selection in this study and others differ considerably. Altamura et al. included consecutive excised lesions (both facial and non‐facial), whereas this study selected a more restricted set of excised non‐facial lesions which were highly suspicious for MM at dermoscopy. Altamura et al. therefore reported different general, frequent criteria, including brown‐black dots/globules, blue‐whitish veil and pigment network, and less frequently atypical vessels. Altamura et al. also concluded that the frequency of melanocytic patterns increased linearly with pigmentation. Our study confirms the presence of the aforementioned MM‐associated patterns, but with very different representations of frequency. Atypical vascular pattern, superficial (short) fine telangiectasia, multiple blue‐grey globules, white‐red structureless areas and white streaks were the most frequently observed features within our cohort, whereas other seven‐point checklist features reported in case reports of BCC diagnosed lesions mimicking MM, including irregular streaks,^10^, ^11^, ^12^ atypical network, blue‐whitish veil and irregular dots/globules,^8^, ^9^, ^10^ were less frequently observed (observations in around 50% of lesions).

White‐red structureless areas^16^, ^17^, ^18^ (observed in over 2/3 of the lesions), white streaks and ulceration^18^, ^19^, ^20^, ^21^ (observed in around half and over 1/3 of the lesions, respectively) are features already reported in literature to be associated with both MM and BCC. Lallas et al. described white‐red structureless areas as a dermoscopic criterion of BCC.^18^ However, it is not a specific BCC criterion, as white and/or red structureless areas are also found in MM (e.g. milky red areas and whitish scar‐like areas).^16^, ^17^ White streaks and ulcerations have also been confirmed common dermoscopic features for both BCCs and nodular MMs.^18^, ^19^, ^20^, ^21^ However, data from this study and others^6^ suggest that atypical vascular pattern should be reconsidered as an MM‐ and BCC‐associated features, and no longer a typical feature of MM only. Future studies are required to better characterize the morphology of atypical vascular patterns in atypical BCC compared to MM.

The presence of both MM‐ and BCC‐associated features in the same lesions render differential diagnosis both difficult and uncertain. The results obtained can improve a differential diagnosis between MM and BCC.^22^ Further, correct in vivo diagnosis can reduce unwarranted patient stress and, as highlighted by Yelamos et al., the considerable level of stress for physicians when evaluating complex, atypical lesions.^23^

The analysis performed in this study was able to identify 12 dermoscopic features, including both MM‐ and BCC‐associated features, which proved helpful in differentially diagnosing between these complex lesions. These features are presented in the diagnostic score provided, which reports high diagnostic sensitivity and specificity. Further, we have also proven that the findings of this study significantly impact clinical diagnostic outcome. The blinded evaluators’ overall diagnostic accuracy and confidence levels were significantly improved after studying the “trump” dermoscopic features we identified.

For further discrimination of lesions, cluster analysis assisted in identifying BCC and MM lesion subtypes, with the main differences between cluster groups being highlighted by the presence or absence of pigment‐related and non‐pigment‐related features. According to these subgroups, the most difficult lesions to diagnose with the lowest physician confidence levels at T0 and T1 were the “hypo/amelanotic” BCC and MM lesions. The main difference between MM and BCC “hypo/amelanotic” lesion clusters was the almost absence of regression structures and multiple small erosions in BCC lesions, which were present in >80% of the MMs. Among all the cluster subtypes, the highest number of misdiagnoses by blinded evaluators were observed among the “hypo/amelanotic” lesions, confirming that the absence of pigment‐related features is the main cause of diagnostic doubt. However, the applicability of this study proved a significant improvement in BCC “hypo/amelanotic‐MM‐like,” but not in MM “hypo/amelanotic” lesions. Diagnostic accuracy and confidence levels for all other BCC and MM subtypes significantly improved.

Correct in vivo differential diagnosis with reduced unnecessary excisions and improved patient management has been proven with the assistance of reflectance confocal microscopy (RCM),^24^, ^25^ and in the case of non‐facial atypical BCC, a diagnostic agreement of 99.5% has been previously reported.^6^ Although RCM was not used in the present study, it is now evident that when available, clinicians should use RCM to enhance preoperative diagnostic performance for equivocal lesions.

This study is limited by its retrospective design and the inclusion of atypical lesions only. The score proposed does not safely select lesions for non‐surgical treatment, as almost 6% of MM are expected to be misdiagnosed as BCC with expert dermatologists. The study did not include a dermoscopic‐pathological correlation analysis, and we did not stratify the score for lesion thickness. Prospective studies are needed to validate the proposed score and further confirm the applicability of these findings in daily clinical practice, among experienced and novel dermoscopy dermatologists and compared with histopathological findings.

This study identified 12 “trump” dermoscopic features for the differential diagnosis among BCCs mimicking MM and MM with BCC features. The score can be used to assist in excision timing but cannot safely be used to select lesions for non‐surgical treatment. Our findings support the notion that atypical vascular pattern should be considered a shared feature of both MM and atypical BCC. Three subtypes of BCC and MM lesions, mainly discriminated by the absence or presence of pigmented features, were associated with different diagnostic accuracies and levels of confidence. The lack of pigmented features and observation of atypical vessels was the most confounding patterns. Further, these findings improve diagnostic accuracy and confidence in daily clinical practice.

## CONFLICTS OF INTEREST

None declared.

## AUTHOR CONTRIBUTION

Eleonora Di Matteo, Francesca Farnetani Riccardo Pampena, Johanna Chester, Shaniko Kaleci, Maria A Pizzichetta, Marco Manfredini, Stefania Guida, Michela Lai, Carmen Cantisani and Silvana Ciardo wrote a part of the manuscript and have made substantial contributions to conception and design, acquisition, analysis and interpretation of data. Elisa Cinotti, Emi Dika, Elvira Moscarella, Aimilios Lallas, Zoe Apalla MD, Giuseppe Argenziano, Jian L Perrot, Linda Tognetti, Vincenzo Roberti, Diletta Fiorani, Carlotta Baraldi, Leonardo Veneziano, Chryssoula Papageorgiou, Pietro Rubegni, Iris Zalaudek, Annalisa Patrizi, Caterina Longo, Luca Bianchi and Giovanni Pellacani involved in the acquisition of data and in revising the manuscript critically for important intellectual content. All authors read and approved the final manuscript.

## ETHICAL APPROVAL

This study is approved by the Ethics committee of Modena (protocol No 169/17).

## Supporting information

Supplementary Material

## Data Availability

The data that support the findings of this study are openly available.
