# Supplementary material for: Unusual dermoscopic patterns of basal cell carcinoma mimicking melanoma
Source: Exp Dermatol. 2022 Feb 6;31(6):890–8. doi: 10.1111/exd.14533 (PMC9305787; doi:10.1111/exd.14533)
Supplement: Supplementary file 1 — Supplementary Material [file EXD-31-890-s001.docx]

**Table S1**. Dermoscopic features classified according to common associations in literature with malignant melanoma (MM) and/or basal cell carcinoma (BCC) diagnoses

| **Revised seven-point checklist** | **Description** | **References** |
| --- | --- | --- |
| **Atypical network** | Combination of at least two types of pigment network (in terms of color and thickness of the lines) asymmetrically distributed within the lesion. | 1 |
| **Blue-white veil** | Irregular. structureless area of confluent blue pigmentation with an overlying white ‘ground-glass’ film. The pigmentation cannot occupy the entire lesion and usually corresponds to a clinically elevated part of the lesion. | 1 |
| **Atypical vascular pattern** | Linear-irregular vessels. dotted vessels and ⁄or milky-red areas not clearly seen within regression structures | 1 |
| **Irregular dots ⁄globules** | More than three round to oval structures. brown or black in color. asymmetrically distributed within the lesion. | 1 |
| **Irregular streaks** | More than three brown to black. bulbous or finger-like projections asymmetrically distributed at the edge of the lesion and not clearly arising from network structures | 1 |
| **Irregular blotches** | Black. brown and ⁄or gray structureless areas asymmetrically distributed within the lesion. | 1 |
| **Regression structures** | White scar-like depigmentation and ⁄or blue pepper-like granules usually corresponding to a clinically flat part of the lesion. | 1 |
| **Dermoscopic structures of BCC** | **Description** | **References** |
| **Arborizing Vessels** | Vessels of large diameter. branching irregularly into finest terminal capillaries. Their color is bright red. being perfectly in focus due to their location on the surface of the tumor. | 2 |
| **Superficial (short) fine telangiectasia** | Short. fine. focused linear vessels with very few branches. | 2 |
| **Blue-gray ovoid nests** | Well circumscribed. confluent or near confluent pigmented ovoid or elongated configurations. larger than globules and not intimately connected to pigmented tumor body. | 2 |
| **Multiple blue-gray globules** | Numerous. loosely arranged round to oval well circumscribed structures. which are smaller than the nests. | 2 |
| **In-focus dots** | Loosely arranged well-defined small gray dots. which appear sharply in focus. | 2 |
| **Maple leaf-like areas** | Translucent brown to gray/blue peripheral bulbous extensions that never arise from pigmented network or from adjacent confluent pigmented areas. | 2 |
| **Spoke wheel areas** | Well-circumscribed radial projections. usually tan but sometimes blue or gray. meeting at an often darker (dark brown. black. or blue) central axis. | 2 |
| **Concentric structures** | Irregularly shaped globular-like structures with different colors (blue. gray. brown. black) and a darker central area. | 2 |
| **Multiple small erosions** | Small brown-red to brown-yellow crusts. | 2 |
| **Dermoscopic feature of both BCC and MM** | **Description** | **References** |
| **Ulceration** | One or more large structureless areas of red to black-red color Loss of the epidermis. usually covered by hematogenous crusts. | 2,3 |
| **White-red structureless areas** | Translucent to opaque white to red areas. | 2-5 |
| **White streaks** | Orthogonal short and thick crossing lines seen only with polarized dermoscopy. | 2-6-7 |

**Table S2**. Demographic and clinical details for the included patients.

|  |  | **Total,**  ***n (%)*** | **BCC,**  ***n (%)*** | **MM,**  ***n (%)*** | **p-value** |
| --- | --- | --- | --- | --- | --- |
|  |  | **222 (100)** | **146 (65.8)** | **76 (34.2)** |  |
| Sex, *n (%)* | Female | 109 (49.5) | 76 (52.1) | 33 (43.4) | 0.312 |
|  | Male | 111 (50.4) | 70 (47.9) | 41 (53.9) |  |
| Mean age* ±*SD (range)* |  | 62.1 ±15.4  (22.1-97.3) | 64.4 ±15.6  (31.1-97.3) | 58.8±14.8  (22.1-83.1) | 0.005 |
| Location. *n (%)* | Trunk | 130 (58.6) | 89 (61) | 41 (53.9) | 0.313 |
|  | Lower limb | 46 (20.7) | 28 (19.2) | 18 (23.7) |  |
|  | Upper limb | 22 (9.9) | 10 (6.8) | 12 (15.8) |  |
|  | Neck | 6 (2.7) | 4 (2.7) | 2 (2.6) |  |
|  | Missing | 18 (8.1) | 15 (10.3) | 3 (3.9) |  |
| Clinical features | Skin phototype: |  |  |  | <0.001 |
|  | I/II | 116 (61.7) | 89 (75.4) | 27 (38.6) |  |
|  | III/IV | 72 (38.3) | 29 (24.6) | 43 (61.4) |  |
|  | Actinic damage in surrounding skin | 109 (58.0) | 69 (58.5) | 40 (57.1) | 0.858 |
|  | Ulceration | 48 (25.5) | 39 (33.1) | 9 (12.9) | 0.003 |
|  | Scales | 42 (22.3) | 34 (28.8) | 8 (11.4) | 0.015 |
|  | Crust | 24 (20.3) | 8 (11.4) | 32 (17.0) | 0.207 |
|  | Shiny surface | 112 (59.6) | 82 (69.5) | 30 (42.9) | 0.001 |
|  | Hair | 35 (18.7) | 6 (5.1) | 29 (41.4) | <0.001 |
|  | Skin markings | 75 (39.9) | 27 (22.9) | 48 (68.6) | <0.001 |
|  | Asymmetry | 105 (55.9) | 55 (46.6) | 50 (71.4) | 0.001 |
|  | Border irregularity | 76 (40.4) | 35 (29.7) | 41 (58.6) | <0.001 |
|  | Color variegation (>2 colors) | 79 (42.0) | 43 (36.4) | 36 (51.4) | 0.004 |
|  | Diameter (>6mm) | 124 (66.0) | 71 (60.2) | 53 (75.7) | 0.030 |

*MM*, malignant melanoma; *BCC*, basal cell carcinoma.

* Age was available for 170 patients only.

**Table S3.** Regression analysis of dermoscopic features predictive for an MM diagnosis (Odds ratio >1) or a BCC diagnosis (Odds ratio <1).

| **Variable** | **Odds Ratio** | **95% Confidence Interval** | **p-value** |
| --- | --- | --- | --- |
| Regression structures | 21.00 | 7.67 - 57.49 | < 0.001 |
| Regular dots/globules | 18.25 | 6.08 - 54.79 | < 0.001 |
| Irregular blotches | 7.00 | 2.60 18.86 | < 0.001 |
| Irregular streaks | 5.34 | 1.32 - 21.61 | 0.019 |
| White-red structureless areas | 4.22 | 1.34 - 13.29 | 0.014 |
| White streaks | 2.48 | 1.05 - 5.84 | 0.037 |
| Spoke-wheel areas | 0.19 | 0.04 - 0.92 | 0.040 |
| In-focus dots | 0.12 | 0.04 - 0.37 | < 0.001 |
| Multiple blue grey globules | 0.11 | 0.04 - 0.29 | < 0.001 |
| Arborizing vessels | 0.08 | 0.03 - 0.21 | < 0.001 |
| Concentric structures | 0.02 | 0.03 - 0.15 | < 0.001 |
| Maple leaf-like areas | 0.02 | 0.00 - 0.08 | < 0.001 |

**Table S4**. Diagnostic accuracy and confidence levels for 2 evaluators, prior to [T0] and following results from this analysis [T1].

|  | **All lesions** | | | **BCC** | | | **MM** | | |
| --- | --- | --- | --- | --- | --- | --- | --- | --- | --- |
|  | **Total,**  ***n (%)*** | **BCC,**  ***n (%)*** | **MM,**  ***n (%)*** | **Hypo/Amelanotic-MM-like,**  ***n (%)*** | **Pigmented-BCC-type,**  ***n (%)*** | **Mixed,**  ***n (%)*** | **Hypo/Amelanotic MM,**  ***n (%)*** | **Pigmented-MM-type,**  ***n (%)*** | **Pigmented-BCC-like.**  ***n (%)*** |
| **Diagnostic accuracy and confidence levels:** | 444 (100) | 292 (65.8) | 152 (34.2) | 87 (29.8) | 65 (22.3) | 140 (47.9) | 21 (13.8) | 74 (48.7) | 57 (37.5) |
| T0, *% (mean level)* | 68.5 (2.9) | 66.8 (2.9) | 71.7 (2.9) | 53.4 (2.7) | 75.8 (3) | 71 (3.1) | 54.2 (2.7) | 82.4 (3) | 64.8 (2.9) |
| T1, *% (mean level)* | 86.9 (3.3) | 90.4 (3.3) | 80.3 (3.3) | 87.5 (3) | 90.9 (3.4) | 92 (3.5) | 54.2 (3.3) | 91.9 (3.4) | 75.9 (3.3) |

*T0*, retrospective blinded evaluator baseline analysis; *T1*, retrospective blinded evaluator analysis following interpretation of results from this study. **References**

1. Argenziano G, Catricalà C, Ardigo M et al. Seven-point checklist of dermoscopy revisited. *Br J Dermatol*. 2011; **164**:785-90.

2. Lallas A, Apalla Z, Argenziano G et al. The dermatoscopic universe of basal cell carcinoma. *Dermatol Pract Concept*. 2014; **4**:11-24.

3. Menzies SW, Kreusch J, Byth K et al. Dermoscopic evaluation of amelanotic and hypomelanotic melanoma. *Arch Dermatol.* 2008; **144**:1120-7.

4. Zalaudek I, Argenziano G, Kerl H et al. Amelanotic/Hypomelanotic melanoma-is dermatoscopy useful for diagnosis*? J Dtsch Dermatol Ges.* 2003; **1**:369-73.

5. Menzies SW, Moloney FJ, Byth K et al. Dermoscopic evaluation of nodular melanoma. *JAMA Dermatol*. 2013; **149**:699-709.

6. Di Stefani A, Campbell TM, Malvehy J et al. Shiny white streaks: An additional dermoscopic finding in melanomas viewed using contact polarised dermoscopy. *Australas J Dermatol.* 2010; **51**:295-8.

7. Liebman TN, Rabinovitz HS, Balagula Y et al. White shiny structures in melanoma and BCC. *Arch Dermatol.* 2012; **148**:146
